# Association of Radiochemotherapy to Immunotherapy in unresectable locally advanced Oesophageal carciNoma—randomized phase 2 trial ARION UCGI 33/PRODIGE 67: the study protocol

**DOI:** 10.1186/s12885-023-11227-0

**Published:** 2023-10-12

**Authors:** Anouchka Modesto, David Tougeron, Pierre Tremolières, Philippe Ronchin, Ariane Darut Jouve, Delphine Argo Leignel, Véronique Vendrely, Olivier Riou, Jérôme Martin-Babau, Samuel Le Sourd, Xavier Mirabel, Thomas Leroy, Florence Huguet, Lucile Montaigne, Isabelle Baumgaertner, Marion Deslandres, Elizabeth Moyal, Catherine Seva, Janick Selves, Philippe Otal, Veronica Pezzella, Rosine Guimbaud, Thomas Filleron, Laurent Quéro

**Affiliations:** 1 Radiation Oncology Department Institut Claudius Regaud at Institut, Universitaire du Cancer de Toulouse-Oncopole, 1 Rue Irene Joliot Curie, 31059 Toulouse, France; 2 Inserm Team 11 RadOpt CRCT 1, Avenue Hubert Curien, 31059 Toulouse, France; 3 Service d’hépato-Gastro-Entérologie, Centre Hospitalier Universitaire de Poitiers, 86000 Poitiers, France; 4 Institut de Cancérologie de L’Ouest: Angers Et Saint Herblain, Saint-herblain, France; 5 Hôpital Privé Arnault Tzanck- Centre Azuréen de Cancérologie, Mougins, France; 6 Institut de Cancérologie de Bourgogne, Dijon, France; 7 Groupe Hospitalier Bretagne Sud, Lorient, France; 8 CHU de Bordeaux, Bordeaux, France; 9 Centre Val d’Aurelle, Montpellier, France; 10 Centre Armoricain d’Oncologie CARIO, Plérin, France; 11 Centre Eugène Marquis, Renne, France; 12 Centre Oscar Lambret, Lille, France; 13 Nouvelle Clinique Des Dentellières, Valenciennes, France; 14 Radiation Oncology Department, Tenon Hospital, AP-HP,, Sorbonne University, Paris, France; 15 Centre Antoine Lacassagne, Nice, France; 16 Centre Hospitalo Universitaire Henri Mondor APHP, Créteil, France; 17 GI Oncology Department Centre Hospitalo, Universitaire Rangueil, Toulouse, France; 18 Pathology department, Centre Hospitalo Universitaire IUCT-Oncopole, Toulouse, France; 19 Imaging Department Centre Hospitalo, Universitaire Rangueil, Toulouse, France; 20 UNICANCER, Paris, France; 21 Biostatistics Departement Institut Claudius Regaud Institut, Universitaire du Cancer de Toulouse-Oncopole, Toulouse, France; 22 Radiation Oncology Department, Saint Louis Hospital, AP-HP, Paris, France

**Keywords:** Esophagus carcinoma, Randomized trial, Phase II, Chemo-radiotherapy, Immunotherapy, Anti-PD-L1

## Abstract

**Background:**

In case of locally advanced and/or non-metastatic unresectable esophageal cancer, definitive chemoradiotherapy (CRT) delivering 50 Gy in 25 daily fractions in combination with platinum-based regimen remains the standard of care resulting in a 2-year disease-free survival of 25% which deserves to be associated with new systemic strategies. In recent years, several immune checkpoint inhibitors (anti-PD1/anti-PD-L1, anti-Program-Death 1/anti-Program-Death ligand 1) have been approved for the treatment of various solid malignancies including metastatic esophageal cancer. As such, we hypothesized that the addition of an anti-PD-L1 to CRT would provide clinical benefit for patients with locally advanced oesophageal cancer. To assess the efficacy of the anti-PD-L1 durvalumab in combination with CRT and then as maintenance therapy we designed the randomized phase II ARION (Association of Radiochemotherapy with Immunotherapy in unresectable Oesophageal carciNoma- UCGI 33/PRODIGE 67).

**Methods:**

ARION is a multicenter, open-label, randomized, comparative phase II trial. Patients are randomly assigned in a 1:1 ratio in each arm with a stratification according to tumor stage, histology and centre. Experimental arm relies on CRT with 50 Gy in 25 daily fractions in combination with FOLFOX regimen administrated during and after radiotherapy every two weeks for a total of 6 cycles and durvalumab starting with CRT for a total of 12 infusions. Standard arm is CRT alone. Use of Intensity Modulated radiotherapy is mandatory.

The primary endpoint is to increase progression-free survival at 12 months from 50 to 68% (HR = 0.55) (power 90%; one-sided alpha-risk, 10%). Progression will be defined with central external review of imaging.

**Ancillary studies are planned:**

PD-L1 Combined Positivity Score on carcinoma cells and stromal immune cells of diagnostic biopsy specimen will be correlated to disease free survival. The study of gut microbiota will aim to determine if baseline intestinal bacteria correlates with tumor response. Proteomic analysis on blood samples will compare long-term responder after CRT with durvalumab to non-responder to identify biomarkers.

**Conclusion:**

Results of the present study will be of great importance to evaluate the impact of immunotherapy in combination with CRT and decipher immune response in this unmet need clinical situation.

**Trial registration:**

ClinicalTrials.gov, NCT: 03777813.Trial registration date: 5^th^ December 2018.

## Background

Esophageal cancer is the 6^th^leading cause of cancer death worldwide with two main histologic subtypes squamous cell carcinoma and adenocarcinoma. Its incidence in France is higher than in any other European country with 5500 new cases in 2018 and carries a dismal prognosis [1].

In case of locally advanced disease and/or non-metastatic unresectable disease, definitive chemoradiotherapy (CRT) delivering 50 Gy in 25 daily fractions in combination with FOLFOX (a combination of oxaliplatin, 5-fluoro-uracil, and acid folinic) administrated during and after radiotherapy every two weeks for a total of 6 cycles is a standard therapeutic option resulting in a 2-year disease-free survival (DFS) of 25% [2]. The majority of patients still die from metastatic progression and the overall benefit of improved local control with optimal CRT remains to be associated with new systemic strategies to prevent metastatic progression.

In recent years, several immune checkpoint inhibitors have been granted approval by agencies such as the US Food Drug Administration (FDA) and the European Medicine Agency (EMA) for the treatment of various solid malignancies including metastatic melanoma, non-small-cell lung carcinoma and esophageal cancer [3, 4]. Last year, anti-Program-Death 1 (anti-PD-1) pembrolizumab has been granted approval by FDA and EMA in first-line of esophageal metastatic disease in combination with platin-based chemotherapy with a PD-L1 combined positive score (CPS) ≥ 10 [5]. Recently in a randomized phase III study (Checkmate 577), the addition of nivolumab for up to one year in the adjuvant setting of resectable esophageal carcinoma with incomplete response after neo-adjuvant CRT resulted in improved median PFS from 11.0 to 22.4 months with a manageable safety profile [6]. In locally advanced esophageal cancer patients PD-L1 expression is correlated with poorer overall survival and its expression in tumor cells increased in esophageal cancer patients after neoadjuvant CRT hence supporting the rationale of PD-L1 blockade in combination with CRT [7].

It is now established that efficacy of chemotherapy and radiotherapy partly relies on the immune system [8]. Combination of immune checkpoint inhibitors and radiation therapy (RT) represents a promising therapeutic strategy supported by a strong pre-clinical rationale [9]. Beyond its local effect, RT induces a rare systemic antitumor response mediated by immune recognition of antigen released from the irradiated tumor known as abscopal effect [10]. Although the primary goal of RT is to achieve local tumor control, ionizing radiations (IR) enhance tumor antigen release that activates immune recognition [11].

The biological mechanisms of the phenomenon called abscopal effect rely on RT to elicit immune tumor recognition that is mediated by various biological mechanisms: recruitment of tumor infiltrating lymphocytes, MHC class 1 antigen presentation in tumor cells that may induced enhancement of tumor antigen presentation or uptake by dendritic cells, cytokines release that facilitates the recruitment of T cells to tumor [12]. PD-1 receptor or its ligand is strongly expressed either on activated T-cells, antigen presenting cells or tumor cells. The activation of these endogenous immune check points play a crucial role to actively evade cytotoxic T-cell activation and immune mediated tumor destruction. The negative regulation of T cells immune response mediated by the PDL-1/ PD-1 axis or the depletion of T cells is known to abrogate RT efficacy [13].

In a randomized phase III study (PACIFIC trial) the addition of anti-Program-Death Ligand 1 (anti-PD-L1) durvalumab for up to 12 months or placebo after definitive radiotherapy up to 66 Gy and platinum-based chemotherapy in unresectable locally advanced non-small cell lung carcinoma showed an increase of median progression-free survival from 5.6 months to 16.8 months and an increase of estimated 5-year rates for OS for durvalumab and placebo were 42.9% versus 33.4% respectively [14].

As such, we hypothesized that the addition of an anti-PD-L1 to CRT would provide clinical benefit for patients with locally advanced oesophageal cancer in increasing local response and preventing metastatic progression. To assess the efficacy of anti-PD-L1 durvalumab in combination with CRT and then as maintenance therapy we designed a randomized 1:1 phase II trial, ARION (Association of Radiochemotherapy with Immunotherapy in unresectable Oesophageal carciNoma ARION UCGI 33/PRODIGE 67). This ongoing prospective intergroup PRODIGE (*Partenariat de Recherche en Oncologie DIGEstive*) open labelled study is supported by Astra Zeneca and sponsored by UNICANCER [15].

## Methods and design

ARION UCGI 33/PRODIGE 67 study is approved by the UNICANCER Gastro-Intestinal Group (UCGI) and by INCa-Labeled cooperative intergroup PRODIGE. It is a French national trial, multicentric, randomized and comparative phase II evaluating the efficacy of durvalumab combined with chemoradiotherapy compared to the standard CRT. The study is conducted in strict compliance with the protocol. Changes will be included in an amended version of the study protocol. Amended trial protocols with substantial modifications are submitted to the ANSM and to the CPP by the sponsor, according to the French regulation.

The Sponsor is responsible to notify to all investigational centers any amendment approved by the French authorities.

### Study objectives

**Fig. 1:**
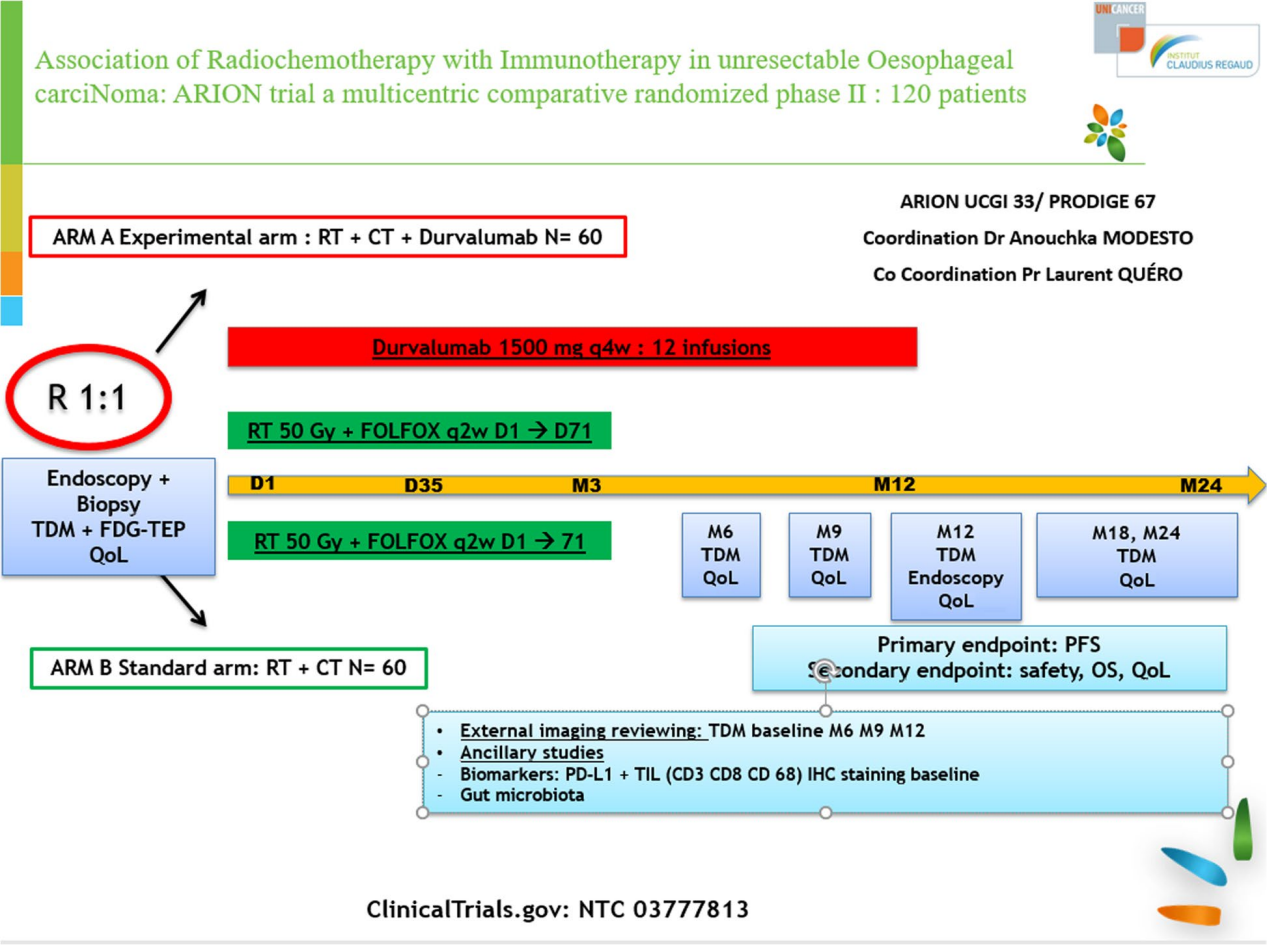
Study design

The primary objective is to assess the efficacy of durvalumab, initially delivered in combination with FOLFOX and IMRT 50 Gy and then as maintenance therapy for treating patients with locally advanced esophageal cancer (adenocarcinoma or squamous cell carcinoma), in terms of centrally reviewed PFS (cPFS). As it is not a placebo-controlled study, an external anonymized blinded independent central review (BICR) of CT-scans (Response Evaluation Criteria in Solid Tumours RECIST v1.1) at baseline, M6, M9, M12, M18, and M24 is planned (see Fig. 1). Secondary objectives are PFS, overall survival, tolerance of the study treatments and quality of life.

Patients fulfilling the inclusion criteria are randomized between the two arms according to a 1:1 ratio using minimization method according to the following factors: *tumor stage, histology and center*.

- ➢ Arm A = experimental arm: durvalumab in combination with FOLFOX and definitive IMRT.
  Chemoradiotherapy (CRT): IMRT 50 Gy in 25 daily fractions in combination with FOLFOX regimen administrated during and after radiotherapy every two weeks for a total of 6 cycles. Durvalumab: 1500 mg one infusion q4w starting with CRT for a total of 12 cycles.
- ➢ Arm B = standard arm: CRT: FOLFOX and definitive IMRT.
  Chemoradiotherapy IMRT 50 Gy in 25 daily fractions in combination with FOLFOX regimen administrated during and after radiotherapy every two weeks for a total of 6 cycles.

Given the lack of safety data from this association, a safety run-in of the 6 first patients was planned to assess for the absence of dose limiting toxicity (DLT).

### Sample size and main statistical analysis

The main objective is to increase 12-months centralized progression-free survival (cPFS) from 50 to 68%. This corresponds to a hazard ratio of 0.55. A total of 74 events expected are necessary for 90% power to detect this difference if it is true using a one-sided logrank test at the 10% level of significance and a 1:1 randomization (arm A: arm B). Target difference, type I and II error rates are compatible with recommendations performed by Rubinstein for comparative phase II trial [16].

Primary endpoint will be analyzed on the Intent-to-Treat (ITT) population when the required number of events is reached. The Kaplan–Meier approach will be used to estimate cPFS rates for each treatment arm. The primary endpoint analysis will be evaluated using a Cox regression analysis with 90% confidence interval (one-sided).

### Patient selection

Inclusion and exclusion criteria are listed in Table 1 (Last version Protocol v5.0_13 Dec 2021).

**Table 1:** List of eligibility criteria of the ARION study

Main inclusion criteria	Main exclusion criteria
• Histologically proven squamous cell carcinoma or adenocarcinoma of the esophagus, • Unresectable disease due to anatomical consideration or medical condition (patient unfit for surgical procedure) according to local multidisciplinary team meeting, • Presence of at least one measurable lesion > 10 mm with spiral CT-scan, • No prior therapy for the esophageal cancer including chemotherapy or radiotherapy prior to the study, except anterior out of field radiotherapy, received for treatment of another primary tumor considered in remission, in the past 5 years, • Age ≥ 18 years old, • WHO performance status < 2, • Body weight > 35 kg, • Patients must have provided consent for the study by signing and dating a written informed consent form prior to any study specific procedures, sampling, or analyses, • Patient must be affiliated to a social security regimen	• Previous treatment with an PD-1, PD-L1 or CTLA-4 inhibitor • Metastatic disease, • Patients should not receive live vaccine 30 days prior to study treatment • Female patients who are pregnant or breastfeeding • Uncontrolled intercurrent illness including, but not limited to diabetes, hypertension, pulmonary failure, chronic renal or hepatic diseases, active peptic ulcer disease or gastritis, active bleeding, diatheses (non-exhaustive list), • Clinically significant cardiac disease or impaired cardiac function, • Current or prior use of immunosuppressive medication within 28 days before the first administration of durvalumab (exception: systemic corticosteroids at physiologic doses not exceeding 10 mg/day of prednisone or equivalent are allowed as well as steroids as premedication for hypersensitivity reactions (e.g., CT scan premedication)—Topical, inhaled, nasal, and ophthalmic steroids are allowed, • Active or prior documented autoimmune or inflammatory disorders • Known primary immunodeficiency or active HIV, • Patient with a dihydropyrimidine dehydrogenase (DPD) deficiency (the test must be done for all patients before 5-FU administration), • Known active or chronic viral hepatitis or history of any type of hepatitis within the last 6 months indicated by positive HBs antibody test for hepatitis B or hepatitis C virus ribonucleic acid (HCV antibody), • Current pneumonitis or interstitial lung disease, • Other invasive malignancy within 2 years prior to entry into the study, except for those treated with curative surgical therapy alone, • History of severe allergic reactions or hypersensitivity to any unknown allergens or any components of the study drug, • Any prior corticosteroid-refractory immune-related adverse event (irAE), • Oeso-tracheal or oeso-bronchial fistulae, • Major surgery within 28 days prior to the first dose of study treatment

### Clinical study endpoints

The primary endpoint is defined by a blinded independent centralized revue of progression-free survival. cPFS is defined as the time from randomization until progression or death; patients alive and without documented progression at last follow-up news have PFS censored at this date or at initiation of new anticancer treatment (if applicable). Progression will be assessed by a blinded independent centralized review of CT-scan per RECIST criteria 1.1.

#### Secondary endpoints are:

- Progression-Free Survival (local PFS) is defined as the time from randomization until progression or deaths; patients alive and without progression at last follow-up news are censored at this date.
- OS defined by the delay between randomization and the occurrence of death due to any cause. Patients still alive at the time of analysis (including lost of follow-up) will be censored at the last known alive date.
- Safety will be assessed by the toxicity grading of the National Cancer Institute Common Terminology Criteria for Adverse Events (NCI-CTCAE v 5.0). To be considered evaluable for safety, patients must have received at least one treatment dose.
- Quality of life (QL) will be assessed by the European Organization for Research and Treatment of Cancer (EORTC) core QL questionnaires, the EORTC QLQ-C30 and QLQ-OES18 (Oesophageal Cancer Module).

### Medical procedures

- FOLFOX 4 simplified protocol, 1 infusion every 2 weeks during 3 months starting with radiotherapy (± 1 day): IV oxaliplatin 85 mg/m2 in 2 h on day 1 (D1), IV Leucovorin 200 mg/m2 in 2 h on day 1 (D1), followed by IV 5-FU 400 mg/m2 in 10 min on day 1 (D1) followed by IV continuous infusion 5-FU 2400 mg/m2 during 46 h.

Premedications use: Anti-emetic premedication and Hematopoietic growth factor will be administered according to local standards of care.

- Durvalumab is delivered to patients included in the experimental arm with a cumulative dose over the whole trial of 18,000 mg (12 cycles):
  ◦ Every 4 weeks during concurrent RT and FOLFOX (dose: 1500 mg): 3 infusions (IV administration before the FOLFOX regimen).
  ◦ Every 4 weeks (dose: 1500 mg) after FOLFOX completion: 9 infusions (IV administration).

### Treatment planning

Radiotherapy will be delivered with FOLFOX with or without durvalumab (depending on randomization) and procedure will be the same for all treated patients randomized in both arms. If feasible, radiotherapy will ideally be delivered after oxaliplatin infusion infusion the days of chemotherapy.

Target volumes are delineated according to standard procedures [17]. Definitive intensity-modulated radiotherapy or helical tomotherapy will be delivered according to boost integrated technique using high-energy photon (≥ 6 MV) medical linear accelerators (linacs) equipped with a multileaf collimator, Volumetric Modulated Arc Therapy (VMAT) or helical Tomotherapy® during 5 weeks, 5 days per week at a dose of:

- 50 Gray (Gy) in 25 fractions of 2 Gy delivered to the macroscopic disease (tumour and involved lymph nodes)
- 45 Gy in 25 fractions of 1.8 Gy to the adjacent peri-tumoural mucosis and prophylactic lymph nodes.

### Quality assurance in radiotherapy

A Quality Assurance (QA) committee for radiotherapy has been implemented for this trial and composed by the coordinating investigators and UNICANCER team, before starting radiotherapy.

For the RT Quality Review, a dedicated database was set up for the study by Aquilab® Company. The quality assurance program for radiotherapy follows the control steps defined by the Quality Assurance Committee (QA committee) provided in the protocol. Each participating center downloads on the database planning and contouring information for all included patients as soon as these data are available. A prospective review is done by the QA Committee, for every first included patient in each participating center and then every 1 patient out of 5 according to a centralized random selection. The RT quality review will take place within 3 working days after download. For the patients concerned, RT planning is validated by the QA Committee before starting treatment. This prospective planning treatment review will provide a statement about the acceptability of the plans and treatment delivery in terms of delineation, dose constraints, and treatment workflow in accordance with the protocol recommendations and will ascertain the absence of any major protocol deviations.

#### Data management

The randomization in each treatment arm will be performed via the UNICANCER data center located in Institut Régional du Cancer de Montpellier – Val d’Aurelle (ICM) Biometrics unit.

All data necessary to the allocation of the treatment arm must be entered timeliness into the study’s case report forms (CRFs). CRFs will be completed by the investigator and other designated members duly designated from his/her staff and will be controlled and validated according to the standard procedures (included those in the software and the sponsor’s quality assurance procedures). When using the eCRF, traceability of access and changes made to the eCRF are traced by the software (audit trail). At the end of the study and once all the eCRF data are validated, the investigator will login to the eCRF to sign all the pages to validate the data entered for each patient.

Each user has received in his personal mailbox an access code (login) and a personal password automatically generated from the plateform to connect to the eCRF. A password non-disclosure certificate is signed by the principal investigator engaging his/her responsibility regarding the confidentiality of the access codes for all users of the eCRF in his/her centre.

To ensure the authenticity and credibility of data in accordance with the “Décision portant sur les Bonnes Pratiques Cliniques, 24 November 2006”, the sponsor establishes a system of quality assurance that consists in:

- The management and the monitoring of the study according to UNICANCER procedures;
- The quality control data of the investigational centers by the monitor involves:
  - verifying that the protocol, as well as the current guidelines ICH-GCP, the national regulatory requirements, are accurately followed,
  - verifying the informed consent and the eligibility of each patient participating in another research,
  - verifying that the CRF data is consistent and in agreement with the source documents,
  - verifying the notification of each SAE,
  - verifying the drug traceability (dispatching, storage and accountability),
  - verifying that patients are not already participating in another research trial which may exclude their inclusion in this protocol. The monitor also verifies that patients have not participate in another trial following which an exclusion period if applicable before they can participate in another protocol,
- The audit of the participating investigational centers when deemed necessary;

The monitors and CRAs in charge of trial monitoring will be mandated by the sponsor. They must have access to all patient data as necessary for their duty in accordance with the national regulatory requirements. The monitors and CRAs are bound by professional secrecy under the national regulatory requirements. Written reports must be issued to ensure monitoring visit traceability. In order to ensure the optimal research quality control, the investigator commits to provide the monitor with direct access to all patient files.

Continuous variables will be summarized by arm, using median, minimum, maximum and number of available observations. Qualitative variables will be summarized by arm using: counts, percents, number of missing data. Primary endpoint will be analyzed on the ITT population when the required number of events has been reached.

All randomized patients signed a written informed consent form. Patient information and informed consent from the patient must be handled in accordance with the “French regulation, especially article L.1122–1 and subsequent articles.

The first patient was enrolled in May 2019 and eighteen centers from the PRODIGE Group are participating. To date, 86 out of 120 patients are already enrolled.

#### Treatment tolerance

Safety is monitored on adverse event (AE) occurrence, the use of concomitant treatments, changes occurring in the course of treatment, observed during physical examination and biological examinations (biochemistry, hematology). Severity is determined according to NCI-CTCAE v5.0.

All AEs that occur from the time of the signing of the informed consent form until 150 days after the last dose of durvalumab, whichever is longer, must be reported by the investigator. All AEs will be recorded on the AE page of the eCRF and in the subject’s source documents.

Deterioration as compared to baseline in protocol-mandated laboratory values and vital signs should only be reported as AEs if the investigator considers the event clinically significant or if they fulfil any of the SAE criteria or are the reason for discontinuation of treatment with the IPs.

If deterioration in a laboratory value or vital sign is associated with clinical signs and symptoms, the sign or symptom will be reported as an AE and the associated laboratory result or vital sign will be considered as additional information. Whenever possible, the reporting Investigator should use the clinical rather than the laboratory term (e.g., anemia versus low hemoglobin value). In the absence of clinical signs or symptoms, clinically relevant deteriorations in non-mandated parameters should be reported as AEs.

Deterioration of a laboratory value that is unequivocally due to disease progression should not be reported as an AE/SAE.

Any new or aggravated clinically relevant abnormal medical finding at a physical examination as compared with the baseline assessment will be reported as an AE. The investigator ensures that adequate medical care is provided to the patient. Treatment of the event may require decoding of the investigational medicinal product.

The investigator must immediately following knowledge of the event, notifies the UNICANCER pharmacovigilance unit of any SAE or any new event defined above, whether or not related to the research, which occurs during the ‘trial reporting period’. This reporting period:

- Starts at the date of the signature on the informed consent form,
- Covers the entire period during which the patient is receiving the investigational treatment or is subject to specific procedures related to the trial,
- Covers a period of 150 days after the last administration of durvalumab for Arm A and 30 days after FOLFOX for Arm B.

Any later SAE, i.e. occurring after a period of 150 days, which is considered to be related to the experimental treatment(s) or to the research (other treatment used, diagnostic procedures and examinations carried out during the research) must be reported without any limitation in terms of deadline.

Notification must be carried out immediately to the UNICANCER pharmacovigilance unit by sending the form “notification of a SAE”, located in the Investigator Master File, completed as precisely as possible, dated and signed by the physician-investigator.

Abnormal laboratory results should be reported as SAE if they possibly put at risk the patient or they require medical intervention to prevent an outcome corresponding to one of severity criteria.

Second cancer, whether or not related to the research, must be reported to the UNICANCER pharmacovigilance unit without any limitation in terms of deadline.

The investigator shall send additional information to the UNICANCER pharmacovigilance unit using a SAE declaration form. All pregnancies and outcomes of pregnancy should be reported immediately to Unicancer except for pregnancy discovered before the study patient has received any study drugs.

Given the lack of safety data from this association, a safety run-in of the 6 first patients recruited in the experimental arm was planned and revealed no unexpected or detrimental toxicities as such, trial was allowed to continue. Control of AEs related to the treatment are made by the sponsor and investigators from each participating center. Additionally, an Independent Data Monitoring Committee (IDMC), with expertise and experience in esophageal cancer, was set up to evaluate safety and tolerability at the end of the safety-run which observed no Dose Limiting Toxicity. The IDMC meet on regular basis (at least once a year). The second IDMC conducted in March 2022 has noticed that the global safety report for the 2 arms is acceptable with regard to risk/benefit balance initially expected.

### Ancillary studies

#### Diagnosis biopsies

Anti-PD1/anti-PDL1 mAbs represent a major paradigm shift in cancer therapy. However, despite the unprecedented efficacy of these innovative treatments, determination of novel biomarker is of paramount interest. Currently, except PD-L1 expression only few parameters have been identified as predictive biomarkers of response of ICIs. In certain tumors subtypes such as esophageal carcinoma, expression PD-L1 Combined Positive Score (CPS) is associated with a higher response rate and survival to anti PD-1 in advanced/metastatic setting [5]. PD-L1 expression on carcinoma cells and stromal immune cells as well as characterization of Tumor Infiltrating Lymphocytes TILs/macrophages infiltrates will be correlated to disease free survival. Centralized review will be performed on formalin fixed paraffin embedded tumor block from diagnostic biopsies to determine an immunoscore. (Pr Selves, Pathology department CHU Toulouse).

#### Gut microbiota

Intestinal microbiota constitutes the largest accumulation of alien organisms (mainly bacteria) present on or in the human body [18]. Accumulating evidence indicates that the proper development of both intestinal and extra-intestinal components of the immune system requires the gut microbiota [19]. Anticancer immune response can be considered as a desirable form of autoimmunity that may be profoundly shaped by the microbiome. These may involve cross-reactivity between microbial and tumor antigens shaping T cell repertoires and/or microbial products stimulating pattern recognition receptors that influence the type and intensity of immune responses. It is of the utmost importance to understand how the microbiome in particularly the gut microflora, impacts natural cancer immunosurveillance in order to shape treatment-induced immune responses [20].

We hypothesized that in esophageal carcinoma as in many other solid tumors metagenomic signature would predict clinical response/resistance and/or immune-related adverse events. Stool samples from patients enrolled in the study are prospectively collected before the instauration of the treatment to perform metagenomic analyses in order identify metagenomics signatures that predict treatment response/resistance and immune-related toxicities.

#### Plasmatic biomarkers

To identify plasmatic novel biomarkers of response to anti-tumor treatment is of paramount interest in locally advanced unresectable esophageal carcinoma patients. Very limited data are available regarding massive features extraction from longitudinal prospective follow-up regarding this medical condition. To date, ARION UCGI 33/PRODIGE 67 trial represent the first randomized clinical trial assessing the efficacy of PD-L1/PD-1 axis inhibition using durvalumab in combination with CRT and then as maintenance therapy. In ARION UCGI 33/PRODIGE 67 trial, frozen plasmatic samples (2 × 7 ml) from consecutive enrolled patients are collected and stored at baseline, second (M2) and twelfth month (M12) after randomization. Future additional studies will correlate baseline plasmatic features to metagenomics characteristics obtained from baseline microbiota. Plasmatic longitudinal analyses will be performed to monitor tumor response in correlation with clinical data.

Tissue blocks from diagnostic biopsies, baseline stool samples and blood samples at screening M2 and M12 are collected for translational research.

## Discussion

The ARION UCGI 33/PRODIGE 67 trial is one of the few on-going trials assessing anti-PD-L1 and CRT in unresectable esophageal carcinoma. Results of the present study will be of great importance to evaluate the impact of immunotherapy in combination with CRT and decipher immune response in this unmet need clinical situation. Mandatory use of IMRT and online prospective quality control of RT planning ensure robustness and reproducibility of RT delivery. The study will yield a large amount of longitudinal imaging and biologic data.

## Data Availability

Data sharing is not applicable to this article. Contact information of the trial sponsor: arion@unicancer.fr.

## Abbreviation list

AE: Adverse event
ARION: Association of Radiochemotherapy with Immunotherapy in unresectable Oesophageal carciNoma
Anti-PD-1: Anti-Program-Death 1
Anti-PD-L1: Anti-Program-Death Ligand 1
BICR: Blinded independent central review
CPS: Combined Positive Score
CRT: Chemoradiotherapy
DLT: Dose limiting toxicity
IDMC: Independent Data Monitoring Committee
IMRT: Intensity-modulated radiotherapy
NCI-CTCAE: National Cancer Institute Common Terminology Criteria for Adverse Events
OS: Overall survival
PFS: Progression-free survival
PRODIGE: Partenariat de Recherche en Oncologie DIGEstive
QA: Quality Assurance
